# Editorial: Synaptic plasticity across the lifespan: mechanisms, adaptation, and vulnerability

**DOI:** 10.3389/fncel.2026.1907668

**Published:** 2026-06-30

**Authors:** Sandra Guidi, Yanis Inglebert, Beatrice Uguagliati

**Affiliations:** 1 Department of Biomedical and Neuromotor Sciences, University of Bologna, Bologna, Italy; 2 Department of Neurosciences, Université de Montréal, Montréal, QC, Canada

**Keywords:** adaptive/maladaptive plasticity, lifespan neuroscience, neural circuit adaptation, neurodegeneration, neurodevelopment, synaptic plasticity

Synaptic plasticity is a fundamental property of the nervous system that enables neural circuits to adapt to development, environmental stimuli, learning, and age-related challenges. Through the continuous modification of synaptic connections, the brain acquires new information, stores memories, and maintains flexibility throughout life (Bliss and Collingridge, 1993; Citri and Malenka, 2008; Holtmaat and Svoboda, 2009). At the same time, plasticity must be tightly regulated. While insufficient plasticity may limit adaptation and recovery, excessive or dysregulated plasticity can destabilize neural circuits and contribute to neurological disease (Magee and Grienberger, 2020; Turrigiano, 2012). Understanding how mechanisms of synaptic plasticity support adaptation while simultaneously creating vulnerability is therefore a central challenge in neuroscience.

The articles included in this Research Topic address this question from complementary perspectives spanning development, synaptic signaling, metabolism, and neurodegeneration. Together, they highlight the dynamic nature of plasticity across the lifespan and emphasize the importance of maintaining a balance between adaptive and maladaptive processes.

A recurring theme emerging from this Research Topic is that the regulation of plasticity changes substantially during development. In their review, Correa et al. focus on the hippocampal CA2 region, a specialized subfield that plays critical role in social memory and hippocampal information processing. The authors describe how mature CA2 neurons possess features that limit excessive excitability, including dense inhibitory circuitry, perineuronal nets, and molecular regulators such as RGS14, PCP4, and STEP. However, these protective mechanisms are incompletely developed during the early post-natal period, creating a window of increased vulnerability. The review proposes that early-life seizures may disrupt CA2 maturation and alter social memory circuit development, potentially contributing to long-term cognitive and behavioral consequences. This work provides an important framework for understanding how age-dependent regulation of plasticity influences brain function.

At the level of cellular and molecular mechanisms, Munsch et al. investigate spike timing-dependent plasticity (STDP) at thalamo-amygdala synapses. STDP is a physiologically relevant form of synaptic plasticity whereby changes in synaptic strength depend on the timing between pre- and post-synaptic activity. The authors demonstrate that timing-dependent long-term potentiation requires endogenous BDNF/TrkB signaling and depends critically on the integrity of cholesterol-rich membrane microdomains. Disruption of lipid rafts abolished the induction of this form of plasticity, highlighting the importance of membrane organization for neurotrophin-dependent signaling. These findings provide insight into the molecular architecture supporting experience-dependent synaptic strengthening and reinforce the central role of BDNF signaling in adaptive plasticity.

While neuronal activity has traditionally been viewed as the primary driver of synaptic plasticity, accumulating evidence suggests that metabolic state also influences the formation and maintenance of neural circuits. Han and Lagerlöf address this emerging area by investigating the role of O-GlcNAc transferase (OGT), a nutrient-sensitive enzyme linking cellular metabolism to protein regulation. Using cultured hippocampal neurons, the authors show that OGT promotes the accumulation of the AMPA receptor subunit GluA1 in dendritic spines, enhances structural markers of synaptic maturation, and increases excitatory synapse number. Importantly, these effects are dependent on neuronal activity, as they are abolished by chronic activity blockade. The study identifies OGT as a key molecular integrator of metabolic and activity-dependent signals and suggests a mechanism through which physiological state influences synaptic connectivity and function. These findings contribute to a growing appreciation that synaptic plasticity emerges from the interaction between neuronal activity and systemic metabolic regulation.

The relationship between plasticity and vulnerability is explored further in the contribution by Kawabata, who proposes a framework for understanding Alzheimer's disease. Rather than considering amyloid and tau pathology as isolated initiating events, the author suggests that excessive or maladaptive synaptic plasticity may represent a key driver of disease progression. According to this view, compensatory remodeling mechanisms that initially support neural function during aging may eventually become dysregulated, leading to network hyperexcitability, aberrant connectivity, and progressive neurodegeneration. By integrating evidence related to amyloid precursor protein (APP) signaling, endosomal trafficking, network dysfunction, and activity-based interventions, the article offers a broader perspective on the relationship between plasticity and neurodegenerative disease. Importantly, it highlights the possibility that therapeutic approaches aimed at restoring the balance between adaptive and maladaptive plasticity could provide novel opportunities for intervention.

Although these contributions address different biological questions and experimental systems, several common principles emerge. First, synaptic plasticity is strongly influenced by developmental stage. Mechanisms that constrain excitability and stabilize neural circuits mature over time, creating critical periods during which neural systems are particularly sensitive to environmental and pathological influences. Second, plasticity depends on the coordinated interaction of multiple signaling pathways, including neurotrophic factors, membrane-associated signaling complexes, metabolic regulators, and neuronal activity. Third, the distinction between adaptive and maladaptive plasticity provides a useful conceptual framework for understanding both normal brain function and disease. Mechanisms that promote adaptation under physiological conditions may become sources of dysfunction when activated excessively or inappropriately.

Another notable aspect of this Research Topic is the integration of multiple levels of analysis. The studies included in this Research Topic span molecular signaling pathways, synaptic organization, neural circuits, behavior, and disease mechanisms. They illustrate the value of bridging traditionally separate areas of neuroscience in order to obtain a more comprehensive understanding of how plasticity is regulated throughout life.

As neuroscience increasingly adopts lifespan approaches, future research will benefit from investigating how mechanisms of plasticity evolve across developmental stages and interact with environmental, metabolic, and pathological factors. Advances in imaging, circuit analysis, single-cell approaches, and molecular profiling will provide new opportunities to understand how adaptive processes are maintained and how they become dysregulated.

In conclusion, the articles assembled in this Research Topic highlight the remarkable capacity of neural circuits to adapt across the lifespan while emphasizing the importance of mechanisms that constrain and regulate this adaptability. By examining synaptic plasticity from developmental, mechanistic, metabolic, and pathological perspectives, these contributions advance our understanding of how neural systems balance stability and flexibility throughout life.

Finally, we sincerely thank all authors who contributed their work to this Research Topic and all reviewers for their evaluations, constructive feedback, and commitment to scientific rigor. We are also grateful to the editorial staff of Frontiers in Cellular Neuroscience for their support throughout the publication process.
